# Metabolic and Proteomic Profiling of Diapause in the Aphid Parasitoid *Praon volucre*

**DOI:** 10.1371/journal.pone.0032606

**Published:** 2012-02-28

**Authors:** Hervé Colinet, David Renault, Blandine Charoy-Guével, Emmanuelle Com

**Affiliations:** 1 Earth and Life Institute ELI, Biodiversity Research Centre BDIV, Catholic University of Louvain, Louvain-la-Neuve, Belgium; 2 Université de Rennes 1, UMR CNRS 6553 Ecobio, Rennes, France; 3 Proteomics Core Facility Biogenouest, INSERM U1085 IRSET, Campus de Beaulieu, Université de Rennes 1, Rennes, France; University of Minho, Portugal

## Abstract

**Background:**

Diapause, a condition of developmental arrest and metabolic depression exhibited by a wide range of animals is accompanied by complex physiological and biochemical changes that generally enhance environmental stress tolerance and synchronize reproduction. Even though some aspects of diapause have been well characterized, very little is known about the full range of molecular and biochemical modifications underlying diapause in non-model organisms.

**Methodology/Principal Findings:**

In this study we focused on the parasitic wasp, *Praon volucre* that exhibits a pupal diapause in response to environmental signals. System-wide metabolic changes occurring during diapause were investigated using GC-MS metabolic fingerprinting. Moreover, proteomic changes were studied in diapausing versus non-diapausing phenotypes using a combination of two-dimensional differential gel electrophoresis (2D-DIGE) and mass spectrometry. We found a reduction of Krebs cycle intermediates which most likely resulted from the metabolic depression. Glycolysis was galvanized, probably to favor polyols biosynthesis. Diapausing parasitoids accumulated high levels of cryoprotective polyols, especially sorbitol. A large set of proteins were modulated during diapause and these were involved in various functions such as remodeling of cytoskeleton and cuticle, stress tolerance, protein turnover, lipid metabolism and various metabolic enzymes.

**Conclusions/Significance:**

The results presented here provide some first clues about the molecular and biochemical events that characterize the diapause syndrome in aphid parasitoids. These data are useful for probing potential commonality of parasitoids diapause with other taxa and they will help creating a general understanding of diapause underpinnings and a background for future interpretations.

## Introduction

In temperate regions, growth and reproduction of ectotherms are restricted to the warmest parts of the year as low winter temperatures decrease the rate of metabolism and life functions and also limit the availability of nutritional resources. In natural conditions, insects respond to seasonal temperature changes through a range of adaptations such as dormancy which includes diapause, a programmed interruption of the development, and quiescence which refers to a transitory interruption of development in response to adverse conditions [1], [2]. Diapause is a dynamic state widespread among insects and other invertebrates such as nematodes, crustaceans and earthworms [3]–[5]. It is characterized by altered behavior, increased energy reserves, reduced metabolism, arrested development and usually increased resistance to environmental stresses [1], [6].

Diapause is a genetically programmed syndrome that is directed neuro-hormonally [7], [8]. It involves a wealth of sophisticated physiological and biochemical adjustments occurring at different biological levels (genes, proteins and metabolites). Diapause does not simply involve silencing of genes expression but it rather evokes the expression of unique sets of genes with specific temporal patterns [6], [9]. Our understanding of the molecular and biochemical basis of diapause has progressed substantially over the last decades [5], [6], [9]–[11]. However, these advances have primarily concerned model organisms which had their genome sequenced and annotated, while physiological background of diapause remains rather limited in non-model species. A comparative study recently found a relative lack of conservation of genes expression during dormancy in *Caenorhabditis elegans*, *Drosophila melanogaster*, and *Sarcophaga crassipalpis*, suggesting that there may be diverse molecular strategies for producing physiologically similar dormancy responses [12]. Despite significant advances, principally on model organisms, we still know very little about the full range of molecular modifications underlying diapause [12], and there is an ongoing need for studies on non-model organisms exhibiting diapause [11]–[13].

In the present study we focused on *Praon volucre* Haliday (Hymenoptera: Aphidiinae), a parasitic wasp that is commercially produced and distributed as an aphid biocontrol agent. Aphidiines represent a group of parasitic insects that can display both an obligatory and a facultative diapause in response to environmental signals [14], [15]. Aphidiines overwinter as pupae in their mummies (i.e. cocoon spined inside the dead host) [15]. In temperate regions where host populations follow seasonal fluctuations of abiotic conditions, diapause is mainly used to synchronize parasitoids population with their host availability [1], [14], [15]. Besides this role, diapause also facilitates survival over harsh periods by affording higher tolerance to unfavorable environmental conditions [1], [15], [16]. In aphidiines, there is a large plasticity in diapause occurrence, only a part of the population enters diapause while the other part remains active or undergoes quiescence during winter [15]–[17]. In addition, diapause duration can be highly variable within a population [17], [18]. These variable patterns are considered as a form of “spreading the risk strategy” ensuring an optimal exploitation of the unpredictable host resources during winter [15], [17]. The ecological aspects of diapause have been widely studied in aphidiines [14], [15] but the biochemical and molecular underpinnings of diapause have not been investigated in this insect group. Understanding all the aspects of diapause has also applied interests in parasitic insects, as it could help mass-rearing protocols through long-term cold storage. However, so far only few practical applications of diapause for cold storage have been reported in parasitoids [19].

Over the past decade, the so-called ‘omics’ techniques have emerged as powerful tools for studying organism–environment interactions. Proteomics and metabolomics are complementary approaches to gene expression profiling but these techniques have the added advantage of studying the real functional molecules compared to transcriptomics. It is often assumed that the presence of transcripts implies that translation also occurred, but clearly that is not always the case [20]. Large-scale studies of proteins are particularly useful since they are not limited by prior assumptions and can, in a single study, reveal fingerprints of a specific molecular adaptation [11], [21]. In addition, metabolites are downstream of both gene transcripts and proteins, and changes in metabolite levels can thus provide biochemical biomarkers of the integrated response of an organism. Moreover, metabolomics is applicable to all species without any prior knowledge of the genome sequence [22]. So far, most molecular studies related to diapause were based on works at genome and transcript levels [23], [24]. In this study we performed 2D-DIGE (Differential Gel Electrophoresis) proteomics and GC-MS (Gas Chromatography-Mass Spectrometry) metabolomics to elucidate the molecular and biochemical changes underlying diapause in the aphidiine wasp *P. volucre*. This complementary approach was carried-out to decipher the underpinnings of diapause and cross-validate our observations using different types of data.

Based on the common characteristics of diapause, we directly tested *a priori* hypotheses about the abundance of some specific metabolites and proteins. Reduction of mitochondrial activity (i.e. rate of ATP production) is key feature of a hypometabolic state such as diapause [3]. We thus expected signs of reduced energy production coinciding with diapause. This could be manifested, for instance, by a reduction of TCA cycle intermediates. Diapausing *P. volucre* mummies display a reduction of metabolic rate [17], but how does intermediary metabolism change with this metabolic depression? Based on earlier studies [12], [25], we expected a larger dependence on glycolytic and gluconeogenic pathways in the diapausing phenotype. Increased stress tolerance is another conserved feature of diapause [5], [12], we thus speculated that proteins promoting stress tolerance, such as molecular chaperones, would be up-regulated in diapausing mummies. Diapausing *P. volucre* store additional lipid reserves [17], another typical characteristic of diapause [3], so we expected metabolism to shift towards conserving lipid reserves. Finally, the synthesis of cryoprotectants coincides with the diapause state in many insect species [2] but there is no report of the involvement of cryoprotective solutes in diapausing aphidiines. We thus tested whether increase in compounds with cryoprotective function (e.g. polyols, sugars or free amino acids) will be associated with diapause in *P. volucre*.

## Methods

### Insect rearing conditions

The green peach aphid, *Myzus persicae* Sulzer (Hemiptera: Aphidinae) was used as a host for parasitoid rearing. Individuals were collected in agricultural fields around Louvain-la-Neuve (Belgium) in 2006. Aphids were reared in 0.3 m^3^ cages on sweet pepper (*Capsicum annuum* L.) under laboratory conditions at 20°C, ±60% RH and long-day conditions (LD 16∶8 h). The parasitoid *P. volucre* was collected in fields at Fleurus (Belgium) in 2009 and was reared in the laboratory under the same controlled conditions.

### Diapause induction

To induce diapause, we used the treatment described by Colinet et al. [17]. Briefly, at the onset of mummification, parasitoids were directly transferred from 20°C to constant 2°C for 7 days in a thermo-regulated cooled incubator (Model 305, LMS Ltd, Sevenoaks, Kent, U.K.) with saturated relative humidity and continuous darkness. This short cold treatment does not affect the survival of *P. volucre* [26]. As expected, a small proportion of the population (c.a. 10%) entered diapause, manifested by an obvious darkening of mummies. In *P. volucre* there is a clear-cut diapause-mediated polyphenism: diapausing mummies are dark brown while nondiapausing ones are clear brown, which makes mummy color a highly reliable marker for diapause in this species [17]. Sets of mummies from both groups (diapausing and nondiapausing) were snap-frozen in liquid nitrogen and kept at −80°C until protein and metabolite extractions. Proteomic and metabolic variations were compared between nondiapausing (ND) clear control mummies and diapausing dark mummies (D).

### Metabolite extraction and derivatization

For both conditions (ND vs D), seven biological replicates, each consisting of a pool of 30 mummies, were used. Each sample was weighed (fresh mass) using a Mettler® micro-balance (accurate to 0.01 mg) before the extractions. The samples were homogenized in 600 µL of cold (−20°C) methanol-chloroform solution (2∶1) using a tungsten-bead beating apparatus (Retsch™ MM301, Retsch GmbH, Haan, Germany) at 25 agitations per second for 1.5 min. Then, 400 µL of ice-cold MilliQwater was added to each sample and vortexed. After centrifugation at 4,000 *g* for 5 min at 4°C, two aliquots of the upper aqueous phase (which contained polar metabolites) were transferred to new chromatographic glass vials: one containing 300 µL of extract and another with 30 µL. The 300 µL aliquot was used to quantify the majority of metabolites, whereas the 30 µL aliquot was used to quantify the few very abundant compounds. Therefore, for each individual sample, two distinct runs of GC-MS were performed. The vials containing the aliquots were vacuum-dried using a Speed Vac Concentrator (MiVac, Genevac Ltd., Ipswitch, England). Samples were then resuspended in 15 µL of 20 mg.mL^−1^ methoxyamine hydrochloride (Sigma-Aldrich, St. Louis, MO, USA) in pyridine before incubation under automatic orbital shaking at 40°C for 90 min. Then 15 µL of N-methyl-N-(trimethylsilyl) trifluoroacetamide (MSTFA; Sigma, #394866) was added to make a total volume of 30 µL and the derivatization was conducted at 40°C for 30 min under agitation. All the derivatization process was automatized using CTC CombiPal autosampler (GERSTEL GmbH and Co.KG, Mülheim an der Ruhr, Germany), ensuring identical derivatization time and process for all samples.

### Metabolomic fingerprinting

The GC-MS system consisted of a Trace GC Ultra chromatograph and a Trace DSQII quadrupole mass spectrometer (Thermo Fischer Scientific Inc, Waltham, MA, USA). The injector temperature was held at 250°C. The oven temperature ranged from 70 to 147°C at 9°C.min^−1^, from 147 to 158°C at 0.5°C.min^−1^, from 158 to 310°C at 5°C.min^−1^, and then the oven remained 4 min at 310°C. We used a 30 m fused silica column (TR5 MS, I.D. 25 mm, 95% dimethyl siloxane, 5% Phenyl Polysilphenylene-siloxane) with helium as the carrier gas at a rate of 1 ml.min^−1^. One microliter of each sample was injected using the splitless mode (25∶1). We completely randomized the injection order of the samples. The temperature of the ion source was set at 250°C and the MS transfer line at 300°C. Detection was achieved using MS detection in electron impact. In the present work, we used the selective ion monitoring mode (SIM) (electron energy: −70 eV), ensuring a precise annotation of the detected peaks. SIM analysis provides more sensitivity than full scan analysis but it only provides information regarding targeted metabolites [27]. We thus only searched for the metabolites that were included in our spectral database, which included 60 pure reference compounds. The peaks were accurately annotated using both their mass spectra (two specific ions) and their retention time. Calibration curves were set using samples consisting of 60 pure reference compounds at levels of 10, 20, 50, 100, 200, 500, 700 and 1000 µM. Chromatograms were deconvoluted using XCalibur v2.0.7 software (Thermo Fischer Scientific Inc, Waltham, MA, USA). Metabolite levels were quantified according to their calibration curves. Arabinose was used as internal standard to account for potential loss during sample preparation and injection. Calculated concentrations were adjusted according to their internal standard. Finally the concentrations were reported according the fresh mass of each sample.

### Protein extraction procedure

For both phenotypes (ND vs. D), four biological replicates, each consisting of a pool of 30 mummies, were used. Mummies were ground to fine powder in liquid nitrogen and precipitated in 10% trichloroacetic acid in acetone for 2 h at −20°C. After centrifugation at 16,000 g for 30 min at 4°C, the pellets were washed three times with 10% (v/v) MilliQwater in acetone with a centrifugation (16,000 g for 30 min at 4°C) between each wash. The pellets were then solubilized in a 20 mMTris buffer pH 7.4 containing 6 M urea, 2 M thiourea and 4% CHAPS. Then, the samples were sonicated on ice with an ultrasonic processor (Bioblock Scientific, Illkirch, France) 6 times for 10 sec, with a 30 sec stop between each step, using a microtip setting power level at 40% pulse duration. The homogenates were centrifuged (16,000 g for 20 min at 4°C) to remove cellular debris, and the supernatants were ultracentrifuged at 105,000 g for 1 h at 4°C. The cytosol protein fractions (supernatants) were stored at −80°C until analysis. Total protein concentration was determined using the Bradford Protein Assay Kit (Biorad, Marnes-la-Coquette, France) according to the manufacturer's instructions.

### Protein labeling procedure and experimental design

2D-DIGE experiments were performed according to a standardized protocol [28]. Fifty micrograms of protein extracts from individual biological replicates of nondiapausing and diapausing mummies were labeled with 400 pmol of cyanine dyes Cy3 or Cy5 (GE Healthcare, Orsay, France), in a reciprocal manner (i.e., dye swapping). Fifty micrograms of combined protein extracts derived from a mix of all samples were labeled with 400 pmol of Cy2 and used as internal standard for the normalization of spot abundances. All steps of the protein labeling procedure were performed in darkness at room temperature. Proteins were incubated with cyanine dyes for 30 min and then the labeling was stopped by incubation with 10 mM lysine for 10 min. Finally the individual Cy2, Cy3 and Cy5 labeling reactions were mixed and run on the same gel. Each gel contained (i) a control nondiapausing sample, (ii) a diapausing sample and (iii) the internal standard sample. Four different replicate gels were performed to allow subsequent statistical assessments.

### Electrophoresis conditions for analytical 2D-DIGE

Prior to electrophoresis, Cy2-, Cy3- and Cy5-mix labeled proteins were incubated in a solubilization buffer (DeStreak™Rehydration solution; GE Healthcare) containing 0.5% Pharmalytes pH 3–10 in a 450 µL final volume, for 1 h at room temperature. The isoelectric focusing (IEF) was performed with pH 3–10 NL 24 cm IPG strips using an IPGphor isoelectric focusing apparatus at 20°C and with 50 µA/strip according to the manufacturer's instructions (GE Healthcare). A maximum voltage of 8,000 V was applied to reach a total of 60 kVh (step 1: 30 V for 12 h; step 2: 100 V for 1 h; step 3: 500 V for 1 h; step 4: 500 to 1,000 V for 1 h; step 5: 1,000 to 8,000 V for 3 h and step 6: 8,000 V for 46,000 Vh). IPG strips were then stored at −80°C until the second dimension. After IEF, the IPG strips were equilibrated for 15 min at room temperature in SERVA IPG-strip equilibration buffer (Serva Electrophoresis, Heidelberg, Germany) containing 54 mM DTT, and then for 15 min at room temperature with the same buffer containing 112.6 mM iodoacetamide. Equilibrated IPG strips were transferred onto a 26×20 cm 12.5% gel casted onto non-fluorescent gel support (Serva Electrophoresis). The protein separation was carried out in 1X anodal and 1X cathodal buffers (Serva Electrophoresis) at 0.5 W/gel during 1 h and 2.5 W/gel overnight.

### Gel scan and image analysis of analytical 2-D gels

Gels were scanned using a Typhoon™ 9400 imager (GE Healthcare) in fluorescence mode. Cy2 images were scanned with a 488 nm laser using a 520 nm emission filter with a bandpass of 40. Cy3 images were scanned with a 532 nm laser using a 580 nm emission filter with a bandpass of 30. Cy5 images were scanned with a 633 nm laser using a 670 nm emission filter with a bandpass of 30. All gels were scanned at a resolution of 200 µm (pixel size). The global fluorescence intensities of the scanned images were normalized by adjusting the exposure times to the average pixel values acquired. The .gel image analysis was performed using the DeCyder software (version 5.01) with a *P*≤0.01 (Student's *t*-test) for the selection of differentially modulated spots. For each protein, a mean variation ratio (i.e., fold change) based on the four replicate gels of each experimental condition was calculated using the DeCyder software.

### Preparative gels and spot picking

Four hundred micrograms of a mix of protein extracts from all ND and D mummies (i.e. internal standard) were loaded on preparative gels and run in the same experimental conditions than those of the analytical gels (see above). After migration, the preparative gels were fixed two times in 15% ethanol, 1% citric acid for 30 min and labeled 1 h with 0.5% LavaPurple in 100 mM sodium borate, pH 10.5. The gels were washed in 15% ethanol for 30 min, acidified in 15% ethanol, 1% citric acid for 30 min and scanned using a Typhoon™ 9400 imager (GE Healthcare) in fluorescence mode at 532 nm laser using a 560 nm long-pass emission filter. The .gel images were analyzed using Decyder software and matched against the spots referenced in the picking list created after the detection of the significantly up- or down-regulated protein signals in analytical gels. The picking list was exported to Ettan spot picker (GE Healthcare) and the spots of interest were automatically excised and transferred into a 96-well plate.

### In-gel digestion

2D gels spots were digested as described previously [29] with minor modifications. Briefly, gel pieces were washed twice in MilliQ water, dehydrated for 15 min in 100% acetonitrile and dried at 37°C during 20 min. Gel pieces were then rehydrated at 4°C for 15 min in a digestion buffer containing 50 mM NH_4_HCO_3_ and 12.5 ng/µl of trypsin (modified, sequencing grade, Promega, Charbonnières, France). The supernatant was then replaced by 30 µl of 50 mM NH_4_HCO_3_ and the samples were incubated overnight at 37°C. Digestion peptides were extracted from gel pieces by several incubation steps: 20 min in 70% acetonitrile/0.1% formic Acid (FA), 5 min in 100% acetonitrile and 15 min in 70% acetonitrile/0.1% FA. At each step the supernatant was collected and pooled to the previous one. Pooled supernatants were evaporated in a vacuum centrifuge in order to have a final volume of 20 µl.

### Protein identification by nano-LC-MS/MS

The nano-LC-MS/MS runs were performed using nano-LC system Ultimate 3000™ (DIONEX - LC Packings, Amsterdam, The Netherlands) coupled on-line to a linear ion trap HCT Ultra P™ Discovery system mass spectrometer (BrukerDaltoniK, GmBh, Germany). The peptides were first concentrated on a 300 µm i.d. ×5 mm precolumn, Pepmap C18 stationary phase, 5 µm, 300 Å wide pore (DIONEX - LC Packings, Amsterdam, The Netherlands). They were then eluted from the precolumn using a gradient from 98% phase A (0.05% FA in aqueous solution) to 90% phase B (0.05% FA in acetonitrile) at a flow rate of 250 nl/min for 75 minutes directly onto a 300 µm i.d. ×15 cm analytical column, Pepmap C18 stationary phase, 5 µm, 300 Å wide pore (DIONEX - LC Packings, Amsterdam, The Netherlands). The instrument was operated in a data-dependant scan mode automatically switching between MS and MS2 with CID (Collision Induced dissociation) fragmentation.

MS/MS data files were processed (Auto MSn find and deconvolution) using the DataAnalysis (version 3.4; BrukerDaltoniK, GmBh, Germany) software. For each acquisition, a maximum of 2000 compounds were detected with an intensity threshold of 200,000 and the charge state of precursor ions was automatically determined by resolved isotope deconvolution. The proteinScape 2.1 software (BrukerDaltonik GmbH) was used to submit MS/MS data to the following databases: NCBI (June 2011, 14481393 sequences) or NCBI restricted to *Nasonia vitripennis* (June 2011, 10637 sequences) using the Mascot search engine (Mascot server v2.2; http://www.matrixscience.com). Parameters were set as follows: trypsin as enzyme with one allowed miscleavage, carbamidomethylation of cysteins as fixed modification and methionine oxidation as variable modifications. The mass tolerance for parent and fragment ions was set to 1.2 Da and 0.5 Da, respectively. Peptide identifications were accepted if the individual ion Mascot scores were above the identity threshold (the ion score is −10*log(*P*), where *P* is the probability that the observed match is a random event, *P*<0.05). In case of ambiguous assignments (one compound fit to more than one peptide), peptide were accepted based on the peptide score, meaning that the peptide sequence with the highest score is accepted. The compilation of identified peptides to proteins was performed with the ProteinExtractor algorithm [30], [31], so that every protein reported was identified by at least one peptide with significant ion Mascot score (above the identity threshold). For every proteins reported in the identification lists, a combined protein score (metascore) was calculated from the peptides scores with the ProteinExtractor algorithm.

### Statistical analysis

Metabolite and protein levels were compared using Student *t*-tests (*α* = 0.01) to determine those that significantly varied between ND and D phenotypes. All metabolites and matched proteins were ranked in a volcano plot according to their statistical *P*-value and their relative difference of abundance (i.e. fold change) [32]. For the metabolites, a PCA was performed on the whole dataset to detect the compounds contributing the most to the structure separation. This analysis was performed using the ‘ade4’ library in the statistical software ‘R 2.13.0’ (R Development Core Team 2008).

## Results

### Metabolic biomarkers of diapause

Separation of polar metabolites from *P. volucre* mummies yielded well-separated peaks and chromatograms were consistent across samples. Among the 60 metabolites included in our library, 48 were detected in the samples. Among these, we found 16 amino acids, 9 sugars, 8 polyols, 11 metabolic intermediates, and 4 amines and diverse metabolites (see Table 1).

**Table 1:** Metabolites detected in the mummified parasitoids *P. volucre* i.

***Free amino acids***	***Polyols***
Valine (Val)	Glycerol
Leucine (Leu)	Erythritol
Isoleucine (Ile)	Xylitol
Proline (Pro)	Ribitol
Glycine (Gly)	Arabitol
Serine (Ser)	Sorbitol
Threonine (Thr)	Inositol
Alanine (Ala)	Galacticol
Phenylalanine (Phe)	***Intermediate acidic metabolites***
Acide glutamique (Glu)	Phosophoric acid
Ornithine (Orn)	Citrate
Lysine (Lys)	Succinate
Asparagine (Asn)	Malate
Tryptophan (Trp)	Fumarate
Glutamine (Gln)	Glycerate
Tyrosine (Tyr)	Glucuronate
***Sugars***	Pipecolate
Fructose	Quinate
Mannose	Ascorbate
Trehalose	Galacturonate
Glucose	***Amines & other metabolites***
Sucrose	Ethanolamine (ETA)
Maltose	Cadaverine (Cad)
Ribose	Putrescine (Put)
Glucose 6-phosphate (G6P)	Glucono delta-lactone (GDL)
Xylose	

i Metabolites abbreviation in parentheses.

**Figure 1:**
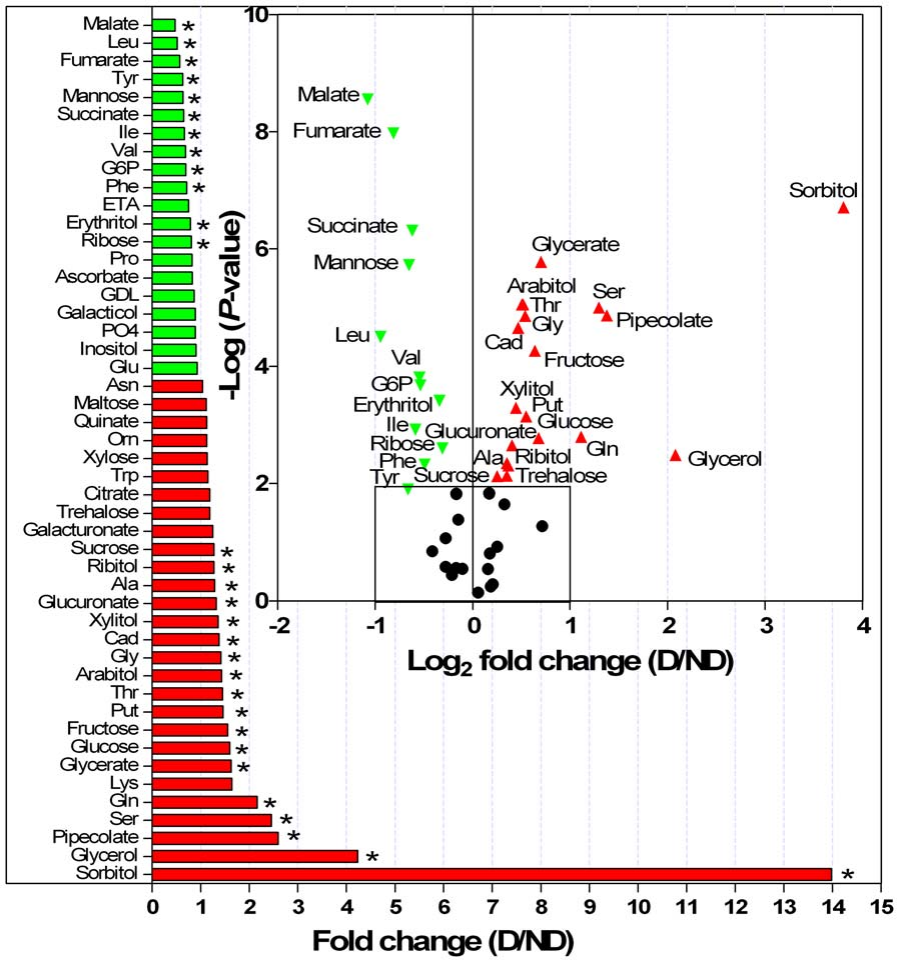
Comparison of metabolite levels in *P. volucre* mummies. Quotients of mean content of diapausing (D) over nondiapausing (ND) are shown (i.e. fold change). Red and green bars represent increased and reduced metabolite levels in D mummies respectively. Stars indicate significant difference between D and ND treatments (*t*-test, *P*<0.05). A volcano plot is enclosed within this figure; metabolites are ranked according to their statistical *P*-value (y-axis) and their relative abundance ratio between D and ND (log_2_ fold change) (x-axis). Off-centred metabolites are those that vary the most between D and ND phenotypes. Symbols (▴), (▾) and (•) for up-regulated, down-regulated and unaffected metabolites in D mummies respectively. Refer to Table 1 for metabolites abbreviation.

About two-thirds of the metabolites identified in this study exhibited significant variation between ND and D phenotypes (Figure 1). Most of these changes, however, were relatively small and only rarely reached a several-fold magnitude. Two polyols, sorbitol and glycerol, were highly accumulated in D mummies (14-fold and 4-fold increase, respectively). A number of metabolites were also more abundant in the D phenotype (Figure 1), among which, pipecolate, Gln and Ser were more than 2*-*fold accumulated in D mummies. Other metabolites, such as fumarate, malate and citrate, were less abundant in D mummies (Figure 1) (about 1.5-2-fold). All the metabolites were represented in a volcano plot (enclosed within Figure 1). On this graph, metabolites located on the left side were on average less abundant, while those located on the right side were more abundant in D mummies.

**Figure 2:**
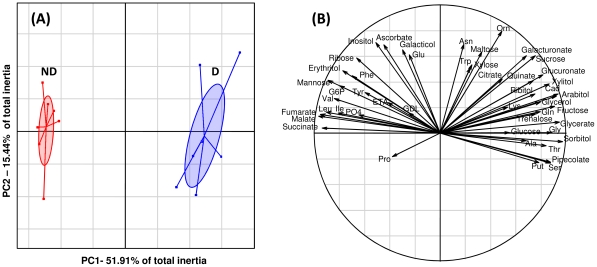
Multivariate analysis (PCA) on metabolomic data. Panel A illustrates that plotting the first two principal components (PCs) results in a clear-cut separation of diapausing (D) and nondiapausing (ND) metabotypes along PC1. Lines link individuals to their respective centroids (*n* = 7). Projection of the 48 variables on the correlation circle is shown in panel B.

A PCA was performed to detect the metabolites that characterized the separation between the two metabotypes. A clear-cut separation was observed along the first principal component (PC1), which accounted for 51.01% of the total inertia (Figure 2A). The metabolites that contributed the most to this separation were sorbitol, glycerate, fructose, glycerol, arabitol, Gly, and pipecolate, and were positively correlated with PC1, while malate, succinate, fumarate, Leu, mannose, Val and G6P were negatively correlated with this axis (see correlation circle, Figure 2B). The other principal components accounted for only 15.44% (PC2) and 10.47% (PC3) of the total inertia and mainly represented within-treatment variations.

### Proteomic biomarkers of diapause

**Figure 3:**
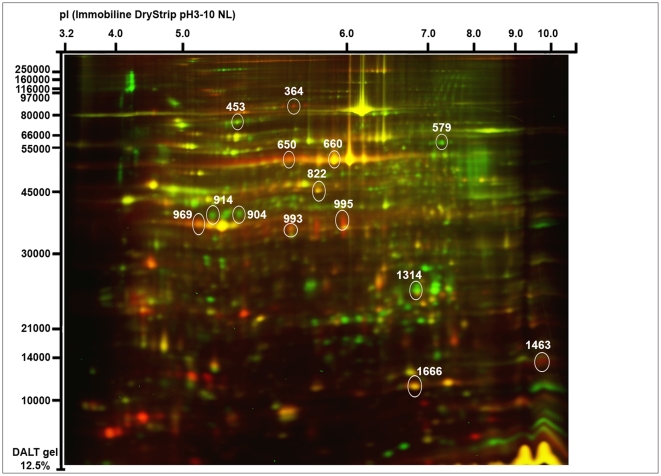
Representative image of the separation of *P. volucre* proteins on a 2D-DIGE gel. On this merged image, the non diapausing group was labeled with Cy3 (green) and diapausing group was labeled with Cy5 (red). Identified proteins showing differential expression level are annotated on the gel with their respective spot number; complete properties of identified proteins are given in Table 2 and Table S1.

**Figure 4:**
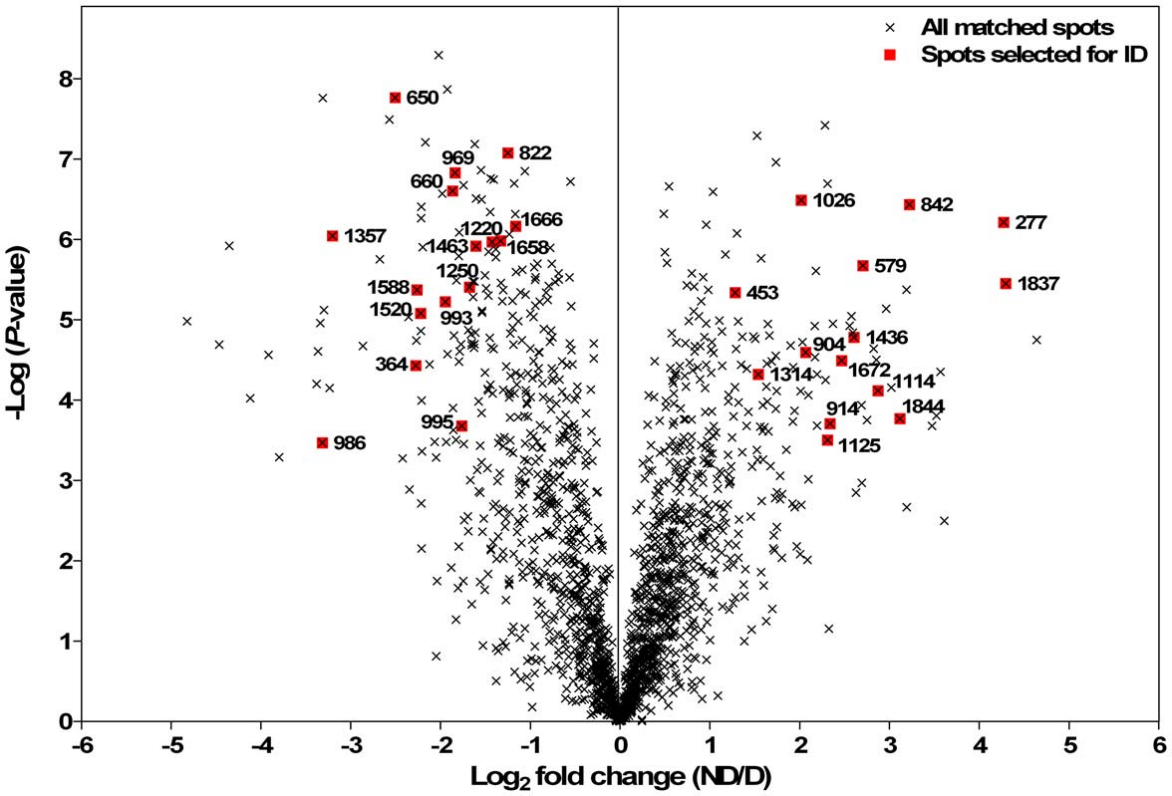
Graphical representation of quantitative proteomics data. Proteins are ranked in a volcano plot according to their statistical *P*-value (y-axis) and their relative abundance ratio (log_2_ fold change) between nondiapausing (ND) and diapausing (D) phenotypes (x-axis). Off-centred spots are those that vary the most between both groups. All matched spots are represented (symbol **×**) together with the 30 spots selected for identification (symbol ▪) with mass spectrometry.

The 2D-DIGE patterns revealed about 1700 matched spots corresponding to *P. volucre* proteome with molecular masses ranging from 10 to 250 kDa, and isoelectric point between 3 and 10. The protein maps showed good reproducibility between the four replicates. A representation is shown in Figure 3. A total of 221 proteins exhibited a significant difference in normalized spot volume ratio exceeding 2-fold between the two phenotypic groups. These differential proteins represented 13% of the total proteome. Proteins were ranked in a volcano plot according to their statistical *P*-value and their relative difference of abundance (Figure 4). Thirty spots were selected for identification based on the magnitude of the response (more than 2 fold), the statistical significance (*P*<0.001), their abundance in the preparative gel, their resolution and the reproducibility among replicates. Spots that were too faint, fused or placed at the border of the preparative gel were not considered. In addition, some spots of interest were discarded as they appeared too faint in the preparative gel. Indeed, the overall protein-staining patterns between LavaPurple image (i.e. preparative gel) and the Cydye images were very similar, but some subtle differences appeared between both types of images. The abundance of some spots in Cy3/Cy5 images can appear either increased or decreased depending on the high/low abundance of lysine composition in the proteins, which is not the case with LavaPurple staining.

**Figure 5:**
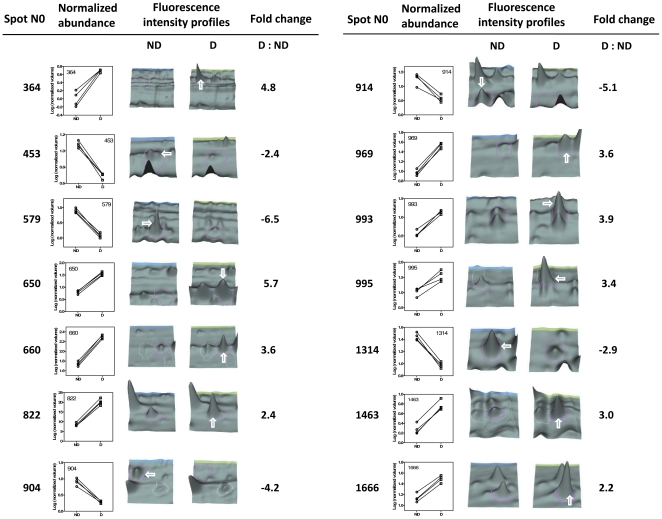
DeCyder output of the identified proteins. Graphs show the normalized spot volumes from four replicate gels for diapausing (D) and nondiapausing (ND) phenotypes, together with three-dimensional fluorescence intensity profiles and corresponding fold changes (D/ND) of the identified spots. Complete properties of identified proteins are given in Table 2 and Table S1.

On the 30 selected proteins, 14 were successfully identified by the mass spectrometry analysis. Identified proteins related to spots numbers are summarized in Table 2, and the differential expressions of these proteins are illustrated in Figure 5. The 14 identified proteins were involved in various biological functions, including cytoskeleton and cuticular component (spots 579, 969, 1314 and 1463), stress response (spots 364), ATP processing (spots 453 and 995), lipid and protein metabolic processes (spots 914, 904 and 993), glycolysis and other metabolic processes (spots 650, 660, 822, 1666). For the spots 993 and 1314, two different peptides matched two different proteins (Table 2). However, in both cases the two identified proteins were of the same family, providing additional support for these identifications. Additional and comprehensive information regarding protein identifications is provided in supplementary Table S1.

**Table 2:** List of significantly modulated proteins identified in *P. volucre* mummies by nano-LC-MS/MS.

Spot No.	Protein name	Score	No. peptides	Source species	Accesion No.	Molecular function	Data base
**364**	PREDICTED: heat shock 70 kDa protein 4L-like isoform1	79.1	1	*Nasonia vitripennis*	gi|345486799	Stress response	NCBI [all]
**453**	PREDICTED: transitional endoplasmic reticulum ATPase TER94-like [Nasonia vitripennis]	1315.7	19	*Nasonia vitripennis*	gi|156548829	ATP binding, nucleoside-triphosphatase activity	NCBI [N. vitripennis]
**579**	PREDICTED: similar to ankyrin repeat protein	35.9	1	*Nasonia vitripennis*	gi|156544844	Cytoskeletal adaptor activity, protein binding	NCBI [N. vitripennis]
**650**	Vesicle amine transport protein	68.6	1	*Bombyx mori*	gi|153792203	Oxidoreductase activity	NCBI [all]
**660**	PREDICTED: similar to GA17549-PA [vanin-like protein]	30.4	1	*Nasonia vitripennis*	gi|156537091	Nitrogen compound metabolic process, hydrolase	NCBI [N. vitripennis]
**822**	Aldehyde dehydrogenase isoform B	209.6	3	*Lysiphlebus testaceipes*	gi|67043753	Oxidoreductase, cellular aldehyde metabolic process	NCBI [all]
**904**	Elongation factor 1-alpha	50.9	1	*Nasonia vitripennis*	gi|307095929	Protein biosynthesis	NCBI [N. vitripennis]
**914**	PREDICTED: apolipoprotein D-like isoform 2	222.8	3	*Apis mellifera*	gi|328786624	Lipid metabolic process, lipid binding, transporter activity	NCBI [all]
**969**	Actin related protein 1	993.8	19	*Nasonia vitripennis*	gi|254910943	Cytoskeleton structure	NCBI [N. vitripennis]
**993**	Cathepsin-L [cysteine peptidase, CP]	69.4	1	*Blattella germanica*	gi|237761900	Proteolysis, cysteine-type endopeptidase activity	NCBI [all]
	Fibroinase [cysteine peptidase, CP]	65.5	1	*Bombyx mori*	gi|164420679	Proteolysis, cysteine-type endopeptidase activity	NCBI [all]
**995**	PREDICTED: similar to arginine kinase-like protein	688.8	12	*Nasonia vitripennis*	gi|156545978	ATP binding, phosphorylation, phosphagen	NCBI [N. vitripennis]
**1314**	Hypothetical protein SINV_09509 [cuticular protein]	121.6	1	*Solenopsis invicta*	gi|322800736	Component of the rigid cuticle	NCBI [all]
	Cuticular protein RR-2 family member 14	82.6	1	*Nasonia vitripennis*	gi|289684255	Component of the rigid cuticle	NCBI [all]
**1463**	PREDICTED: similar to putative muscular protein 20	93.8	2	*Nasonia vitripennis*	gi|156551940	Actin binding, muscle protein, regulation of cell shape	NCBI [N. vitripennis]
**1666**	GD10219 [Glyceraldehyde 3-phosphate dehydrogenase]	183.8	2	*Drosophila simulans*	gi|297673190	Glycolysis, gluconeogenesis	NCBI [all]

## Discussion

### Cryoprotectant biosynthesis

In this study, we combined metabolic profiling and proteomics to study the physiological underpinnings of diapause in the aphid parasitoid *P. volucre*. The synthesis of cryoprotective compounds is supposed to coincide with diapause in many insect species [2]. However, there was so far no report of the involvement of cryoprotective solutes in diapausing aphidiines. We thus tested whether increase in compounds with cryoprotective function (e.g. polyols, sugars or some free amino acids) will be associated with diapause in *P. volucre*. A number of amino acids were more abundant in D mummies. But most of these changes were relatively small which makes their effective contribution to cryoprotective functions unlikely.

Among the metabolites with assumed cryoprotective functions, the most notable changes found concerned two polyols, sorbitol and to a lesser extent glycerol, which accumulated massively in D mummies. Glycerol is by far the most common polyol used by overwintering insects but other polyols include sorbitol, mannitol, ribitol, xylitol, erythritol [33]. Single or multicomponent polyol systems have been described in insects 34,35. In diapausing eggs of the silkworm, *Bombyx mori*, sorbitol and glycerol also accumulate at high concentration [36]. It is generally accepted that sorbitol protects from heat [37] and cold injuries [38] in insects. The accumulation of polyols in D mummies, mainly sorbitol, thus likely represents a physiological adaption to potential unfavorable thermal conditions. Accumulated cryoprotectants can produce a colligative depression of melting and supercooling points, but it is becoming clear that compatible solutes, such as polyols, can protect cells in various ways other than osmotically [35], for instance by stabilizing membranes and macromolecules [38], [39]. Both glucose and fructose could be used as precursors for sorbitol synthesis [40], [41]. This could explain why both metabolite levels increased slightly in D mummies. It is generally assumed that exposure to cold triggers cryoprotectant synthesis [42]. In our experimental design, all individuals were exposed to cold but only D mummies accumulated polyols. Therefore it appears that the ability to accumulate polyols is specific to diapause state.

### Energy metabolism

In *Pieris brassicae*, the concentration of sorbitol is closely correlated with the level of diapause-induced metabolic suppression [43]. An evolutionary scenario suggested that polyols accumulation is a byproduct of diapause-induced metabolic suppression [44]. Reduction of metabolic rate is indeed a typical feature of insect diapause [45], and this was also observed in D mummies of *P. volucre* [17]. Consequently, we expected signs of reduced mitochondrial energy production coinciding with diapause. We found that the level of TCA cycle intermediates (malate, succinate and fumarate) significantly decreased in diapausing mummies. Likewise, another metabolomics approach recently found a reduction in pools of aerobic metabolic intermediates during diapause [25]. This metabolic response, which appears to be shared among different species, likely resulted from the reduction of TCA cycle activity. The same authors also noted an increase of pyruvate during diapause [25]. The increase of pyruvate is not surprising as there must be an inhibitory block of glycolysis at this specific locus to favor polyols synthesis rather than TCA cycle [40]. Indeed, production of polyols requires a regulatory control of glycolysis and pentose cycle in order to divert the carbon flow from the main stream [40], [46]. In the present study, we found an up-regulation of glyceraldehyde 3-phosphate dehydrogenase (GAPDH) in D mummies (spot 1666). The up-regulation of GAPDH, an enzyme playing a crucial role in glycolysis, corroborates the notion that diapause increases glycolysis [12], [25]. A proteomic study of diapause in the parasitoid *N. vitripennis* also found up-regulation of GAPDH [11]. This suggests that GAPDH might be an important regulatory point of glycolytic flux for polyols synthesis, as suggested by Storey [40]. The observed reduction of mannose level in D mummies (−1.6 fold) may result from its transformation into fructose/glucose equivalents for use in the glycolytic pathway [25] or sorbitol synthesis, although this need further testing. Overall our data support the growing consensus that diapause increases glycolysis and gluconeogenesis, and decreases aerobic metabolism, probably to facilitate metabolic depression, stress tolerance and synthesis of cryoprotectants [12], [25].

### Stress tolerance

Increased stress tolerance is a conserved feature of diapause [5], [12], we thus speculated that proteins promoting stress tolerance, such as molecular chaperones, would be up-regulated in diapausing mummies. We found that a heat shock protein 70 (spot 364) was more abundant in D mummies. Molecular chaperones are involved in diverse functions including transport, folding, unfolding, assembly, disassembly, and degradation of misfolded or aggregated proteins [47]. The up-regulation of HSPs during dormancy is a common pattern across insect species [12], [23], [48] and our data confirm this view. Aldehyde dehydrogenase (ALDH) was up-regulated in D mummies (spot 822). This metabolic enzyme is essential for physiological homeostasis and it also catalyzes the oxidation of toxic compounds providing protection against environmental stresses such as oxidative and osmotic stress [49]–[51]. Up-regulation of ALDH has also been found in other diapausing arthropods [11], [52], [53], but its causative role in diapause remains to be established. Except from polyols whose protective functions have been described above, pipecolate was also accumulated in D mummies (2.6-fold). This metabolite has osmoprotective capacities [54], [55] and could thus contribute to stress tolerance. However, whether this function underlies its up-regulation also requires further investigations.

### Cytoskeleton and cuticular components

Several proteins related to cytoskeleton structure were differentially modulated between ND and D mummies. An actin protein (spot 969) was up-regulated in D mummies. Up-regulation of actins during diapause is a common response in insects [10], [11], [56], but down-regulation of actin (proteins or transcripts) has also been reported [24], [57], [58]. Actins constitute a large family of proteins, with specific localization and regulation; therefore different actins might be elevated while others might decrease concomitantly during diapause [56]. Changes in the polymerization of actin have been clearly established during diapause [59], suggesting that remodeling of actin cytoskeleton has important role during diapause. Other cytoskeleton-related proteins were modulated. An ankyrin repeat protein (spot 570) was down-regulated, while a muscular protein 20 (MP20) (spot 1430) was up-regulated in D mummies. Ankyrins comprise a family of ubiquitously expressed membrane adaptor molecules that play important roles in coupling integral membrane proteins to the cytoskeleton network [60]. Ankyrin repeat motifs are found in many proteins and their functions include the maintenance of cytoskeleton integrity [61]. MP20 is a muscle-specific protein that has a developmental pattern similar to that of other muscle-specific proteins, such as actin and tropomyosin [62]. Collectively our observations corroborate the notion that cytoskeletal reorganization accompanies diapause syndrome.

A cuticular protein (spot 1314) was down-regulated in D mummies. Cuticle is a dynamic structure that is reorganized during the preparative phase of insect diapause [3]. Structural constituents of the cuticle are generally over-represented in diapausing stages [24], [63]. Like actins, cuticular proteins constitute a very large family [64], [65], some might be up-or down-regulated concurrently during diapause. Thus the differential expression seen here strengthens the idea that the protein composition of diapausing insects cuticle is distinct from that of nondiapausing insects [66]. Together, the altered expressions of cytoskeleton, contractile and cuticular proteins suggest a restructuring of these compartments in response to diapause in *P. volucre*.

### Lipid metabolism and protein turnover

It was previously shown that diapausing mummies of *P. volucre* accumulate lipids in preparation for diapause [17]. We thus expected metabolism to shift towards conserving lipid reserves. We found a down regulation of an apolipoprotein-D (spot 914), a member of the lipocalins family [67], which serves as a lipid transporter [68]. Down-regulation of a gene encoding apolipoprotein-D was also found in diapausing *Helicoverpa armigera* [69] and changes of lipid metabolism with diapause was noted in *N. vitripennes* [11]. The down-regulation of a lipid carrier is consistent with lipid sparing of diapause.

We found a down-regulation of Elongation factor 1-alpha (spot 904), an essential component of the translational machinery [70]. A down-regulation of Elongation factor 1 was also found in other diapausing insects [11], [69]. Since this protein promotes protein biosynthesis, a down-regulation may indicate that translation is suppressed (or reduced) during diapause, which is consistent with the notion of developmental arrest during diapause. Along the same line, Reynolds and Hand [71] reported that a number of transcripts related to protein synthesis, including Elongation factor 1, were significantly depressed in diapausing cricket embryos.

A cysteine peptidase (spot 993) was more abundant in D mummies. Cysteine proteases play important roles in extracellular and intracellular proteolysis in a wide range of organisms [72]. In insects, they have various functions, including protein degradation during digestion [73]. Cysteine peptidases were also abundant in diapausing *Artemia sinica* [74] and transcription of genes encoding a variety of proteolytic enzymes is up-regulated during reproductive diapause of *D. melanogaster* [63]. The buildup of proteolytic digestive enzymes during developmental and reproductive arrest is likely to pave the way for resumption of digestion upon diapause termination [5], [75].

### ATP-binding proteins

A protein corresponding to arginine kinase (AK) (spot 995) was up-regulated in D mummies. AK is the sole phosphagen kinase found in several major invertebrate groups, including arthropods and molluscs [76]. Members of this enzyme family play key roles as ATP-buffering systems in animal cells that display variable rates of ATP turnover [76], [77]. The up-regulation of this metabolic enzyme, in early *P. volucre* diapause may, at first, seem counterintuitive as diapause involves a reduction of energy production. Indeed, down-regulation of AK (transcript or protein) has been reported in diapausing ectotherms [24], [69], [71]. However, other studies also found that AK was abundant during diapause [74], [78]. This discrepancy probably arises from the fact that temporal expression of AK greatly varies during the early phases of diapause. Temporal expression of AK has recently been studied, and it appeared that its level was high in early diapause before declining progressively to reach undetectable level 25 days following diapause onset [78]. The high abundance of AK in *P. volucre* is thus consistent with this temporal pattern, as proteomic variations were assessed in the early phase of diapause.

A transitional endoplasmic reticulum ATPase (TER94-like) (spot 453) was down-regulated in D mummies. This protein was also less abundant in diapausing cotton bollworm [79]. TER94 is a member of the AAA family of ATPases [80] and is the insect orthologue of vertebrates TERs. Members of this family are necessary for the fragmentation of golgi stacks during mitosis and for their reassembly after mitosis [81]. Since reduction (or cessation) of mitosis characterizes diapause, it is coherent that the expression of this protein was down-regulated during diapause.

### Concluding remarks

So far, most molecular studies related to diapause were based on works at genome and transcript levels [23], [24]. In this study we performed 2D-DIGE proteomics together with metabolic fingerprinting, in order to elucidate the molecular and biochemical changes underlying diapause in a non-model aphid parasitoid. We found a reduction of TCA cycle intermediates which likely resulted from the metabolic depression of diapausing parasitoids. Glycolysis was galvanized, probably to favor cryoprotective polyol biosynthesis, especially sorbitol which greatly accumulated in diapausing parasitoids. As far as we are concerned, this is the first report of the involvement of cryoprotective polyols in aphidiines. Results from proteomic and transcriptomic studies suggested that diapause-related polyols synthesis likely occurs through the acceleration of glycolysis [11], [12], [69], however metabolite levels had never been assayed concurrently to clearly establish this statement. The present work provides such connection. We also found a large set of modulated proteins during diapause; these were involved in various functions such as remodeling of cytoskeleton and cuticle, stress tolerance, protein turnover, lipid metabolism and various metabolic enzymes. These data will help creating a general understanding of diapause underpinnings and a background for future interpretations.

The diversity of organisms showing diapause and the variety of developmental programs recognized as dormant suggest that several properties of diapause may vary from species to species [5]. The results presented here provide some first clues about the molecular and biochemical events that characterize diapause syndrome in aphid parasitoids. The genome of a parasitic wasp, *N. vitripennis*, has recently been added to the Hymenoptera Genome Database (HGD) [82], however, the protein database for Hymenopteran insects is still incomplete and many protein functions still remain unknown. Quantitative protein analysis thus remains challenging, especially for non-sequenced model species such as *P. volucre*. This research provides a basis for deciphering diapause characteristics in an ecologically-important parasitoid species and will be useful for probing the potential commonality of diapause with other taxa. More research needs to be performed to match the identity of the cryoprotective compounds in other diapausing species, and identify the functions of proteins involved in this syndrome.

## Supporting Information

Table S1 **List of the identified proteins.** The following information is displayed in the table: Spot Numb (Decyder master gel spot number); Cmpd. (DataAnalysis compound number), m/z meas. (m/z measured), Mr calc. (Mr calculated), z (charge), Δ m/z (m/z deviation) [ppm], RMS90 (root mean square) [ppm], Rt (retention time) [min], scores (individual mascot ion score), Peptide sequence (sequence of the identified peptide), Accession (NCBI accession number), Protein (Protein Name).
(XLSX)
